# Psychosocial and Functional Predictors of Mental Disorder among Prostate Cancer Survivors: Informing Survivorship Care Programs with Evidence-Based Knowledge

**DOI:** 10.3390/curroncol28050334

**Published:** 2021-10-03

**Authors:** Lia Massoeurs, Gabriela Ilie, Tarek Lawen, Cody MacDonald, Cassidy Bradley, Jasmine Dang Cam-Tu Vo, Robert David Harold Rutledge

**Affiliations:** 1 Department of Community Health and Epidemiology, Dalhousie University, Halifax, NS B3H 1V7, Canada; Lia.Massoeurs@dal.ca (L.M.); CodyMacDonald@dal.ca (C.M.); cs724384@dal.ca (C.B.); 2 Department of Urology, Dalhousie University, Halifax, NS B3H 4R2, Canada; Tarek.Lawen@Dal.Ca; 3 Department of Psychology and Neuroscience, Dalhousie University, Halifax, NS B3H 4R2, Canada; Jasmine.Vo@dal.ca; 4 Department of Radiation Oncology, Dalhousie University, Halifax, NS B3H 4R2, Canada; Rob.Rutledge@nshealth.ca; 5 Faculty of Medicine, Dalhousie University, Halifax, NS B3H 1V7, Canada

**Keywords:** mental disorder, prostate cancer, survivorship, urinary problems, quality of life, emotional well-being, social/family well-being, spiritual well-being, functional well-being

## Abstract

Recent research has revealed that prostate cancer (PCa) survivors are facing a silent epidemic of mental disorder. These findings are not surprising when the side effects of highly effective current treatment modalities are considered. Here, we assess the association between urinary function and quality of life indicators to mental disorder among survivors of PCa. This is a cross sectional examination of an analytical sample of 362 men with a history of PCa residing in the Maritimes who took a survey assessing social, physical and health-related quality of life indicators between 2017 and 2021. Mental disorder was assessed using Kessler’s Psychological Distress Scale (K-10). Predictor variables included emotional, functional, social/family and spiritual well-being, measured by Functional Assessment of Cancer Therapy-Prostate (FACT-P), and urinary function was measured by International Prostate Symptom Score (IPSS). Multivariate logistic regression analysis evaluated the contribution of predictors while controlling for age, income, survivorship time (months) since diagnosis, relationship status and treatment modality. Mental disorder was identified among 15.8% of PCa survivors in this sample. High emotional (aOR = 0.81, 95% CI: 0.69–0.96) and spiritual well-being (aOR = 0.88, 95% CI: 0.81–0.96) were protective factors against mental disorder. Men who screened positive for moderate to severe urinary tract symptoms had three times higher odds (aOR = 3.02, 95% CI: 1.10, 8.32) of screening positive for mental disorder. Men who were on active surveillance or radical prostatectomy with or without added treatment had higher (aOR = 5.87, 95% CI: 1.32–26.13 or aOR = 4.21, 95% CI: 1.07–16.51, respectively) odds of screening positive for mental disorder compared to men who received radiation treatment with or without hormonal therapy for their PCa diagnosis. Unmet emotional and spiritual needs, increased urinary problems and some forms of treatment (e.g., active surveillance or surgery) were associated with mental disorder among PCa survivors. The development of survivorship care programs and support systems that focus on the long-term effects of PCa treatments and the consequences of unmet psychosocial needs of patients during the survivorship journey are critically needed.

## 1. Introduction

Approximately 64 men each day are diagnosed with prostate cancer (PCa) in Canada [1]. Along with a diagnosis of PCa comes an extensive list of physical and closely linked psychosocial stressors predisposing PCa patients to the development of poor mental health [2,3,4,5,6,7,8,9]. According to Fervaha et al. (2019), approximately 1 in 6 PCa patients experience depression, which has been shown to affect both quality of life and oncologic outcomes [2]. Recent research based on large population samples has revealed that men who have a history of a PCa diagnosis have double the odds of depression and anxiety compared with men who have never had a PCa diagnosis [4,10]. Men with a history of PCa also have 1.5 times higher odds of screening positive for depression and anxiety compared with survivors of any other form of cancer [7]. Although treatments for localized PCa are highly effective, research finds that PCa survivors report numerous survivorship stressors, including urinary problems, sexual and intimacy concerns due to erectile dysfunction, lack of emotional support, isolation, loneliness, fatigue, sleep problems, treatment regret, and very poor (less than 20%) attendance to PCa or any other type of cancer support groups [3,5,8,11,12,13,14]. Younger age, low income (less than CAD 50,000), smoking, increased alcohol misuse, relationship dissatisfaction, multimorbidity, having a pre-existing clinical diagnosis of depression or anxiety, and high neuroticism have all been identified as psychosocial markers for mental disorder among PCa survivors [6,7,8,10,11,15]. The psychiatric implications of a PCa diagnosis and follow up through active and non-active forms of treatment on oncological outcomes and quality of life have only been recognized for their magnitude in recent years. In a recent meta-analysis by Brunckhorst et al. (2021), 117 studies were reviewed, and it was found that 5.81% of PCa survivors (representing 655,149 patients in 11 studies) had depressive disorders, 17.07% had significant depressive symptoms (32,339 patients in 76 studies), 16.87% had significant anxiety symptoms (24,526 patients in 56 studies), 9.85% reported suicide ideation (6,173 patients in 8 studies), and the crude mortality rate after diagnosis was 47.1 per 100,000 persons per year in 12 studies [16]. 

Given the longevity of survivorship post-localized low grade PCa (minimal risk of dying from PCa, 20 years later), it becomes important to identify these psychosocial determinants of mental disorders in the post-treatment period. With early identification of at-risk patients comes timely evidence-based interventions to prevent the development of mental disorders [17]. Traditionally, researchers and clinicians alike attributed most mental health issues to the physical sequelae of PCa treatments (e.g., genitourinary symptoms, bowel issues, etc.). Only recently have we begun to consider the contribution of psychosocial determinants to the emergence of mental disorders in PCa patients [2,4,5,6,10,15].

Research has shown that, when compared to PCa patients with mild or no urinary symptoms, patients with moderate to severe urinary symptoms have a five-fold increased risk in screening positive for mental disorder (OR = 4.69; 95% CI: 1.04–22.03) [5,6]. Among all treatments, men who underwent surgery for PCa were found to have a seven times higher risk of post-treatment depression when compared to those undergoing any other form of therapy [8]. Similarly, lower socioeconomic status was associated with a ten times higher risk of screening positive for depression as compared to higher socioeconomic status [7]. Taken together, these results indicate that biological, psychological and social variables significantly contribute to the onset of depression among men with PCa [2,15,16]. 

Fervaha et al. (2019) highlight the importance of identifying risk factors for depression among PCa survivors, as psychological and social variables are often overlooked [2]. There is evidence abound that all patients suffering from an array of chronic illnesses benefit from quality emotional and spiritual supports [18]. For example, in a study of HIV patients, Brady et al. (1999) found spiritual well-being to be as important a contributor to the health-related quality of life model of HIV as several physical factors [19]. Research has also shown that PCa patients commonly suppress emotions related to their disease, subsequently leading to anger, worsened quality of life and avoidance of emotional support [20]. Of note, spirituality has been shown to improve an individual’s mental health through social support, meaning-making and self-regulation [21,22]. Social support from family, friends and other loved ones is critical factor in helping PCa patients cope with the diagnosis, treatment and long-term effects of their disease [23,24,25].

To our knowledge, this is the first study to assess the inter-related contributions of psychosocial well-being, treatment modality and quality-of-life physical factors (e.g., urinary function) to the mental health outcomes of men with PCa. Our study derived data from a population-based sample of men with a history of PCa in the Maritime provinces in Canada. These analyses were controlled for age, income, relationship status, and survivorship time elapsed since diagnosis.

## 2. Methods

This study analyzed 362 men (mean age = 68.55 years) diagnosed with localized PCa who were surveyed between May 2017 to January 2021 as part of a Canadian Maritime provinces survey examining the quality of life of PCa survivors. Participants eligible for this study had to reside in the Maritimes, have had a history of a diagnosis of localized PCa, were English speakers, and had a valid email address. Eligible patients were identified through PCa support groups, urology and radiation oncology clinics and were provided a link to the survey. Among the patients who were provided a survey link, 68% responded. The duration of the online survey was approximately 25 minutes. Patients were able to access the study online through their own electronic devices, or a device was able to be provided to them in these settings. The study data were kept in an online database through the REDCap web application (Research Electronic Data capture) supported by the regional health authority. All participants provided consent which was obtained electronically at the time of survey access. Survey procedures were in accordance with the ethical standards of the responsible committee on human experimentation and with the Helsinki Declaration of 1975, as revised in 2000. The study was approved by the Nova Scotia Health Authority Research Ethics Board (# 1021455).

### 2.1. Measures

#### 2.1.1. Outcome

Mental disorder. The Kessler Psychological Distress Scale (K10), a well-validated, ten-item Likert-style survey that screens for the presence of mental disorder [26]. The K10 categorizes each score from an overall range of 10 to 50, with a score of 10–19 indicating likely to be well (no presence of mental disorder), 20–24 indicating mild mental disorder, 25–29 indicating moderate disorder, and above 30 indicating severe mental disorder [27]. No mental disorder (scores below 20) was coded 0, and the presence of mental disorder (scores 20 or above) was coded 1. Cronbach’s α for K10 in our sample was 0.89, which is comparable to the reliability coefficient (0.88–0.91) reported elsewhere [28,29].

#### 2.1.2. Predictors

Social/family, emotional and functional well-being were predictors, and were assessed using the Functional Assessment of Cancer Therapy-Prostate (FACT-P) [30]. The social/family and functional well-being subscales consist of 7 items each and the emotional well-being subscale consists of 6 items, with these items being rated from 0 to 4 (0 = “Not at all”, 1 = “A little bit”, 2 = “Somewhat”, 3 = “Quite a bit”, 4 = “Very much”). Items for social/family well-being assessed the participant’s closeness to friends, emotional support from family, support from friends, acceptance of the PCa illness by family, satisfaction with family communications, closeness to partners and main supporters and satisfaction with sex life. Emotional well-being items addressed feeling sad, level of satisfaction of coping with PCa, feelings of nervousness, worry about dying and worry about the PCa condition worsening. Functional well-being items assessed ability to work, fulfilment of work, enjoyment of life, acceptance of the PCa diagnosis, ability to sleep well, enjoyment of the things that they usually do for fun and how content they are with their quality of life. The total scores for each domain were formed by summing the score from each item to create a range from 0–28 for social/family and functional well-being, and 0–24 for emotional well-being. One item in the emotional well-being domain was reverse coded before being included in the total score. For all sub-domains and total scores, greater scores indicated greater well-being and greater quality of life [31]. 

The FACT-P also assessed a physical well-being domain, which was administered but was not included in the analysis in order to avoid multicollinearity with the urinary predictor measure (I-PSS) we have included in the model. The Cronbach’s alpha for the social/family, emotional and functional well-being domains assessed in our sample was 0.84, 0.74, and 0.88, respectively. These indicate overall good internal reliability and are comparable to those reported in the literature [32,33].

Spiritual well-being was assessed using the extended version of Functional Assessment of Chronic Illness Therapy- Spiritual Well-Being (FACIT-Sp12 Version 4), which consists of 12 questions and assesses three independent subdomains: meaning, faith and peace [18]. Each question offers five responses ranging from 0 to 4, (0 = “Not at all”, 1 = “A little bit”, 2 = “Somewhat”, 3 = “Quite a bit”, 4 = “Very much”). The meaning items addressed having a reason for living, productive life, sense of purpose and lack of meaning and purpose. Peace items addressed feeling peaceful, trouble feeling peace of mind, deep self-comfort and feeling a sense of harmony within oneself. Faith items addressed finding comfort in faith of spiritual beliefs, finding strength in faith or spiritual beliefs, illness strengthening faith or spiritual beliefs and knowing that with whatever happens with illness that things will be okay. The questions “I have trouble feeling peace of mind” and “My life lacks meaning and purpose” were reverse coded before scoring. Sum scores for spiritual well-being ranged from 0 to 48. Higher scores indicated better spiritual well-being. FACIT-Sp12 has a range of reliability coefficients in the literature, with a Cronbach’s α from 0.72–0.87 [34]. Cronbach’s α for FACIT-Sp12 in our analytical sample was 0.81.

Urinary tract symptoms were assessed through the International Prostate Symptom Score (I-PSS), which is well validated and commonly used in clinical settings for rapid diagnosis and track of urinary tract symptoms [35,36,37]. I-PSS assesses the urinary tract symptoms experienced by PCa patients or survivors to track and suggest their medical management [35]. Urinary symptoms addressed by the items in the questionnaire are incomplete emptying of the bladder, frequency of urination, intermittency of urination, urgency of urination, weakness of urinary stream, straining to urinate and nocturia. Responses for each of the items range from 0—“not at all”; 1—“less than 1 in 5 times”; 2—“less than half the time”; 3—“about half the time”; 4—“more than half the time”; to 5—“almost always”. The absence or presence of mild urinary problems is indicated by summing the scores for all items. Scores from 0–7 were coded 0, while the presence of moderate (sum scores ranging from 8–19) or severe (sum scores ranging from 20–35) problems were coded 1. Cronbach’s α for I-PSS in this sample was 0.85 which is comparable to the scale’s reliability reported in the literature (0.88) [38].

Treatment modality. Participants were asked to indicate if they received either radical prostatectomy, radiation (external beam or brachytherapy) or hormonal therapy (injections, pills or orchiectomy) for their PCa diagnosis, or if they were currently on active surveillance. To ensure a minimum cell count, treatment modality was coded as 0 for active surveillance, 1 for radical prostatectomy with or without additional forms of treatment and 2 for radiation therapy or hormone therapy alone (or both). 

Covariates. Age, past year household income (coded 1 for < CAD 50,000, 2 for CAD 50,000–CAD 100,000, 3 for > CAD 100,000), current relationship status (coded 0 for widowed, single, or divorced or 1 for currently in a relationship, married, common-law or dating/seeing someone at the moment) and months elapsed between diagnosis and survey were all covariates in the final analysis (multivariate logistic model). The choice of coding for current relationship status was to ensure minimum cell count.

### 2.2. Statistical Analyses

Analyses were performed using SPSS V27. Prior to conducting the analyses, the assumptions of logistic regression were checked and found to be tenable. Cross-tabulations and individual logistic regression analyses were used to assess the association between mental disorder and the six predictors (urinary, social/family, emotional, functional and spiritual well-being, treatment modality) and 4 covariates (age, relationship status, household income and survivorship time (months) since diagnosis). A multivariate logistic regression analysis was used to model the outcome based on the predictors and the covariates. The primary outcome had 21.5% of missing data. Little’s MCAR test was statistically significant (*p* < 0.001), indicating that data were not missing at random. To determine if the results obtained were contingent on how the missing data was handled, sensitivity analyses were conducted. Although a visual examination of the missing data did not reveal any systematic patterns, multiple imputation (MI) was used to supplement the analyses and add confidence to the results obtained. MI was performed using SPSS V.26, using an iterative Markov Chain Monte Carlo (MCMC) algorithm known as fully conditional specification (FCS) or chained equations imputation. The number of imputations, 41, was randomly generated to represent a value within the range of 33 to 100, as recommended by the literature [39,40]. The multivariate logistic regression model included the pooled MI results to assess comparison tenability. After listwise deletion, the analytical sample for the multivariate logistic regressions was 284.

## 3. Results

A total of 15.8% of men screened positive for the presence of mental health issues. The mean age of respondents was 68.55 years (±7.12 years), with an average time between diagnosis and completion of the survey of 63.23 months (±57.73 months). The majority of men in this sample had a household income in the past year of up to CAD 100,000 (69.8%), and almost all men in the sample reported currently being in a relationship at the time of the survey (80.4%). Most men in this sample received active treatment(s) for their PCa diagnosis (72.9%) and were retired or unemployed at the time they were surveyed (72.3%). Among the men in the sample, 37.9% reported moderate to severe urinary tract symptoms. The means and standard deviations for the social, emotional, functional and spiritual well-being are presented in Table 1 along with the rest of the demographic characteristics of the sample. 

Logistic regression analyses assessed the relationship between each of the predictors and covariates and the outcome, mental disorder. The presence of moderate to severe urinary tract symptoms (OR = 3.00, 95% CI: 1.51–5.97) and the lack of active treatment for the PCa diagnosis (active surveillance, OR = 2.97, 95% CI: 1.14–7.71) were positively associated with mental disorder. Low social/family well-being (OR = 0.83, 95% CI: 0.78–0.88), emotional well-being (OR = 0.70, 95% CI: 0.63–0.78), functional well-being (OR = 0.80, 95% CI: 0.74–0.85) and spiritual well-being (OR = 0.84, 95% CI: 0.80–0.88) were associated with the presence of mental disorder among PCa survivors. Increased age (OR = 0.95, 95% CI: 0.91–0.99) was a protective factor for mental disorder. Lastly, survivors on active surveillance had almost three times higher odds of mental disorder compared with survivors who received radiation treatment with or without hormone therapy (OR = 2.97, 95% CI: 1.14–7.71). No other statistically significant effects between predictors or covariates and the outcome variable emerged (see Table 2).

A multivariate logistic regression model assessed the presence of screening positive for mental disorder and examined the contribution of the six predictors (severity of urinary tract symptoms, treatment modality, social/family well-being, functional well-being, emotional well-being and spiritual well-being) and the four demographic covariates (age, household income, survivorship time, current relationship status) and was found to be statistically significant, X^2^(12) = 90.23 *p* < 0.001, with Nagelkerke’s R^2^ = 0.54 and the Hosmer and Lemeshow test showing model stability X^2^(8) = 2.497, *p* > 0.05 (see Table 3). This model was accurate for nearly 87.9% of the individuals in this sample. When all other predictors were held constant, the adjusted odds were 5.87, 95% CI: 1.32–26.13 (OR_MI_ = 4.95, 95% CI: 1.55–15.82) and 4.21, 95% CI: 1.07–16.51 (OR_MI_ = 3.95, 95% CI: 1.15–13.63), times higher for screening positive for mental disorder among men who were on active surveillance or who received radical prostatectomy with or without added treatment for their PCa diagnosis, respectively, compared to those who received radiation therapy with or without added hormonal therapy. Men who had moderate or severe urinary tract symptoms had 3.02, 95% CI: 1.10–8.32 (OR_MI_ = 3.27, 95% CI: 1.38–7.75), times higher odds of screening positive for mental disorder compared with those with mild or no urinary tract symptoms, when all other variables in the model were held constant. Both low emotional and spiritual well-being were associated with the presence of screening positive for mental disorder (aOR = 0.81, 95% CI:0.69–0.96; aOR = 0.88, 95% CI: 0.81–0.96, respectively or aOR_MI_ = 0.87, 95% CI:0.76–0.98; aOR_MI_ = 0.92, 95% CI: 0.86–0.99) in the full adjusted model. Results from the pooled MI model were comparable.

## 4. Discussion

During the PCa journey, a number of physical, emotional and psychological challenges can emerge for patients. These challenges can emerge from the several types of treatment modalities used for PCa, each with their own significant side effects. At several points along the trajectory, men with PCa face several stress-inducing decisions, starting with prostate biopsy, choice of treatment modality, post-treatment recovery, treatment-related sequelae and, finally, how to respond to a rising prostate-specific antigen (PSA) level after treatment. Some patients may also have to face some physical changes that they otherwise they may not have had to face until later in life (e.g., the possibility of losing erectile function). PCa recurrence after primary surgery or radiation therapy also poses numerous challenges. 

Here, we examined the impact of urinary tract symptoms, treatment management, and psychosocial well-being on the mental health of PCa survivors while controlling for demographic variables. To our knowledge, this is the first study to assess the association model of mental disorder using these combined predictors and covariates. Results indicate that low emotional and spiritual well-being, moderate to severe urinary tract problems, as well as the presence of active surveillance or surgery (with or without other treatment modalities) compared with radiation therapy were statistically significantly associated with mental disorder, when each of the other variables in the model were held constant. 

These results corroborate existing evidence. Previous studies have found that men with PCa express fear when facing diagnosis, PSA test results and with worsening sexual and urinary function [41,42,43]. Long-term side effects of treatment include incontinence, erectile dysfunction, urinary symptoms (e.g., frequency, dysuria), bowel irritation, libido decline, weight gain, hot flashes and fatigue [44,45,46].

The results in our study show that radiation therapy with or without hormone therapy was a protective factor for mental disorder compared with those on active surveillance or surgery (radical prostatectomy) which had almost six (aOR = 5.87, 95% CI: 1.32–26.13) and almost four (aOR = 4.21, 95% CI: 1.07–16.51) times higher odds of mental disorder, respectively. Our results align with a recent Atlantic Canadian study which found that men undergoing surgery for PCa had a more than seven-fold risk of depression compared to men undergoing surgery for any other form of cancer [8]. Side effects from surgery, particularly when combined with other active treatments (i.e., radiation and/or hormone therapy) can be associated with anxiety and depression [2,5,6,15,16]. Survivors of PCa may express loss of hope for a cure, regret for earlier treatment choices, belief that other treatment options may have been superior, or fear of their ultimate prognosis [11]. Studies have shown that psychological distress is prevalent in both PCa survivors (47%) and their partners (>75%) [47].

Together, these results indicate that treatment modality has a significant impact on the psychological well-being of a PCa survivor. The existing body of evidence can be conflicting on this point. Ravi et al. (2014) found that active PCa treatment was a protective factor against the onset of depression, anxiety, or suicide in a population of elderly men [48]. However, a 2015 systematic review found no major negative impact to health-related quality of life or psychological well-being in men on active surveillance [49]. Low self-efficacy, or one’s perceived ability to conquer stress challenges and adapt, as well as personality type (particularly high neuroticism/low emotional stability), may be factors associated with the emergence of mental health illness while undergoing active surveillance or surgery [3,50,51]. Research shows that breast cancer survivors with high self-efficacy report positive psychosocial adjustment, emotional, social, and physical well-being, as well as better quality of life [51]. 

Lazarus and Folkman (1984) describe the dilemma with active surveillance and self-efficacy by comparing problem-focused versus emotion-focused coping [52]. For example, men undergoing definitive PCa treatment feel in control, with their treatment acting as the solution to their problem (problem-focused coping) [52,53]. When compared to active treatment, men undergoing active surveillance feel less in control of their disease, or less self-efficacy, which can result in increased anxiety and psychological distress [51,53]. To mitigate feelings of no control, patients can use emotionally focused strategies (emotion-focused coping) and seek social support [51,53]. 

Another finding from our study is the statistically significant and independent association between moderate-to-severe urinary symptoms and mental disorder. Urinary dysfunction is a common side effect of PCa treatment, particularly with surgery, and can significantly impact the long-term emotional well-being of PCa survivors [6,45,54]. Our results show that men with moderate-to-severe urinary problems had a three times higher risk of screening positive for mental disorder (OR = 3.02, 95% CI: 1.10–8.32). Similarly, Gillis et al. (2021) reported a five times higher risk of mental disorder with worse urinary symptoms (OR = 5.21, 95% CI: 1.94–14.05) [3]. Moreover, Orom et al. (2018) found that among PCa patients treated with surgery or radiation, worsening urinary, sexual or bowel function were all associated with emotional distress [55]. Boevé et al. (2021) quantified the issue and found that a significant proportion of PCa patients reported statistically significant worsening of bowel and urinary symptoms in the first 6 months after treatment [56].

Lastly, our results indicate that a PCa patient’s low emotional and spiritual well-being were independently associated with poor mental health. These results are novel but consistent with the literature. Spirituality has been reported by PCa survivors to be beneficial for coping with all aspects of PCa care [57]. Patients with chronic disease were found to have lower quality of life when they scored low on the spiritual well-being scale of FACT-Sp [58]. Similar results were reported for PCa patients by Krupski et al. (2006), who found that survivors of PCa who were spiritual had better health-related quality of life than those who were not spiritual. Traeger et al. (2009) measured post-treatment emotional well-being in PCa patients using the Functional Assessment of Cancer Therapy-General (FACT-G) [54]. They found that high emotional well-being was associated with higher levels of perceived treatment control, higher levels of perceived comprehension of one’s condition, lower levels of perceived negative impact of PCa on one’s personal life, and fewer perceived personal and behaviour risk factors for developing PCa, such as “my diet or eating habits” or “my attitude and personality”. Spirituality is often ignored by those of a non-religious background as it is commonly used interchangeably with the term religion—which is erroneous. Religion is defined as the institutionally sanctioned beliefs and activities of a particular faith group [59]. Spirituality, on the other hand, may be synchronous with religion, but can exist without adherence to a religious belief and vice versa [59,60,61]. Zavala et al. (2009) found that PCa patients reporting high spirituality had significantly less urinary and bowel bother, greater urinary and bowel function, and greater overall health-related quality of life [62]. Unfortunately, the literature states that a significant percentage of PCa patients report having little to no spiritual support from the medical system, which highlights the need for improved awareness in this domain. Patient spirituality has been shown to be important, with its potential to shape an individual’s health-related quality of life, psychosocial outcomes and ultimately, oncological outcomes [62,63,64]. 

Depression, anxiety, and other forms of mental disorder are often overlooked in clinical care for PCa patients. Depression can be masked by symptoms of cancer and its treatment, as the symptoms are similar, with patients experiencing fatigue and loss of appetite and/or sleep [65]. Traditional oncologic care is neglectful of mental illness and survivorship needs, with the focus on immediate oncological outcomes and physical rehabilitation [2,4,16]. Studies have found that many providers are unaware of the psychosocial, spiritual and emotional needs specific to PCa survivors, leading to a lack of follow-up for these specific issues [66]. With the lack of a comprehensive and coordinated follow-up care program, PCa survivors do not have their holistic needs met; this in turn negatively impacts their oncological outcomes and quality of life [65]. Cancer survivorship care plans are increasingly popular, with a comprehensive focus on pre- and post-treatment rehabilitation [67,68,69]. Recent patient education and empowerment programs are now emerging, providing education and empowerment to men diagnosed with PCa. These programs focus on evidence-based lifestyle modifications to promote improved quality of life and reduced mental health illness [67]. Together, these results indicate the need for the implementation of mental health screening for PCa patients and survivors as part of their oncologic care plans [2,16,67]. 

The current study is not without limitations. All data from the study are self-reported and based on volunteer participation. Our results represent associations; therefore, causal inferences cannot be made. Our data is subject to recall bias. Our sample size was relatively small and included men with relatively high survivability; thus, survival bias is a potential concern. Therefore, the results may lack generalizability to all populations of PCa patients with various degrees of illness severity. Future studies with larger sample sizes using a mixed-method design should consider evaluating the contribution of other demographic variables known to be associated with mental disorder. These mental illness risk factors include ethnicity, socio-economic status, cancer recurrence and co-morbidities. Future studies of a larger sample size should also attempt to examine the possibility of interactive effects between, for example, urinary function and treatment type (as some treatments are more likely to affect urinary function than others). To ensure minimum cell count for the analysis, current relationship status, treatment for PCa and employment status were coded in smaller categories than we would have preferred. Future studies with larger sample sizes should consider looking at differences between various relationship status, treatment types, and employment status categories. Lastly, our choice of dichotomization of the outcome variable was based on severe positive skewness which did not improve post transformation. However, as Streiner (2002) points out, dichotomizing continuous data results in loss of information and statistical power. Future studies should attempt to maintain the continuous scores when possible, given the potential limitations of this practice [70]. 

Despite these limitations, our results have merit and extend the existing literature. The insights gained from these data add a unique perspective to the existing body of literature. We found that being treated with surgery or non-active treatment (active surveillance), moderate to severe urinary tract symptoms and poor emotional and spiritual well-being significantly contribute to mental disorder amongst PCa survivors when all other predictors and covariates are held constant in the model. Given the lack of prospective data evaluating mental health risk factors among PCa survivors, our data sheds light on the vulnerability of these patients and the dire need for multidisciplinary survivorship care plans. Given the recent evidence highlighting the silent epidemic of mental disorder among PCa survivors, the development of patient education and empowerment programs to assist these men is of utmost importance [67].

## 5. Conclusions

The present study investigated the association between treatment modality, psychosocial well-being, urinary symptoms and mental disorder in PCa survivors. The results extend the existing literature, which emphasizes the importance of screening for mental disorder in PCa patients and the dire need for survivorship care programs. Our results suggest that clinical, community and social engagement are integral to the survivorship of PCa patients. 

## Figures and Tables

**Table 1:** Demographic characteristics of a sample of men with a history of prostate cancer diagnosis from the baseline cycle of a Quality-of-Life Maritimes Survey administered between 2017 and 2021, *n* = 362.

Variable	Sample Characteristics
**Age** (n, *Mean* (SD, range))	321, *68.55* (7.117, 47–88)
**Education** (n, %)	
Highschool or less	42, 14.5%
Bachelor or less	166, 57.4%
Graduate education	81, 28.0%
**Currently in a relationship** (*n*,%)	291, 80.4%
**Retired or unemployed**, (*n*,%)	232, 72.3%
**Household income** (*n*,%)	
<50K	73, 25.1%
50K–100K	130, 44.7%
>100K	88, 30.2%
**Survivorship time (months) from diagnosis** (n, *Mean*, (SD))	316, *63.23* (57.73)
**Treatment modality** (*n*,%)	
Active surveillance	90, 27.1%
Surgery (alone or plus other forms of treatment)	140, 42.2%
Radiation or Hormone therapy alone, or both	102, 30.7%
**Urinary tract symptoms**, **I-PSS**, (*n*,%)	
None to mild	203, 62.1%
Moderate or severe	124, 37.9%
**Social/family well-being ^1^**, **FACT-P** [n, *Mean* (SD, min, max score)]	362, *20.02* (5.52, *0–28*)
**Emotional well-being ^1^**, **FACT-P** [n, *Mean* (SD, min, max score)]	362, *19.98* (3.90, *1–24*)
**Functional well-being ^1^**, **FACT-P**, [n, *Mean* (SD, min, max score)]	361, *20.92*, (5.50, *0–28*)
**Spiritual well-being ^1^**, **FACIT-Sp**, [n, *Mean* (SD, min, max score)]	359, *32.91*, (9.36, *5–48*)

^1^ Lower scores indicate worse quality of life.

**Table 2:** Cross-tabulations assessing the relationship between the outcome (mental disorder), predictors (urinary function, social/family, emotional, functional and spiritual well-being, treatment modality), and covariates in a sample of men with a history of prostate cancer diagnosis from the baseline cycle of a Quality-of-Life Maritimes Survey administered between 2017 and 2021, *n* = 284.

Predictors	Screening Negative for Mental Disorder (*n* = 239) *OR (95% CI)*	Screening Positive for Mental Disorder (*n* = 45) *OR (95% CI)*	Wald X^2^
**Urinary tract symptoms**, **I-PSS**, %			X^2^(1) = 9.78 **
None to mild	62.5% 1.0 Reference	35.7% 1.0 Reference	
Moderate or severe	37.5% 1.0 Reference	64.3% 3.00 (1.51–5.97) **	
**Social/family well-being ^1^**, **FACT-P** [Mean (SD)]	21.31 (4.77) 1.0 Reference	15.16 (6.19) 0.83 (0.78–0.88) ***	X^2^(1) = 34.46 ***
**Emotional well-being ^1^**, **FACT-P** [Mean (SD)]	21.06 (2.83) 1.0 Reference	16.04 (4.51) 0.70 (0.63–0.78) ***	X^2^(1) = 42.85 ***
**Functional well-being ^1^**, **FACT-P**, [Mean (SD)]	22.29 (4.72) 1.0 Reference	15.60 (5.40) 0.80 (0.74–0.85) ***	X^2^(1) = 40.41 ***
**Spiritual well-being ^1^**, **FACIT-Sp**, [Mean (SD)]	35.26 (8.16) 1.0 Reference	22.73 (8.16) 0.84 (0.80–0.88) ***	X^2^(1) = 45.03 ***
**Treatment Modality**, %			X^2^ (2) = 5.02
Active surveillance	13.9% 1.0 Reference	26.2% 2.97 (1.14–7.71) *	
Surgery (alone or plus other forms of treatment)	48.6% 1.0 Reference	50.0% 1.62 (0.72-3.63)	
Radiation or hormone therapy alone, or both	37.5% 1.0 Reference	23.8% 1.0 Reference	
**Age**, [Mean (SD)]	69.13 (6.82) 1.0 Reference	66.49 (8.67) 0.95 (0.91–0.99) *	X^2^(1) = 5.01 *
**Survivorship time (months) from diagnosis**, [Mean (SD)]	66.46 (58.40) 1.0 Reference	62.30 (55.45) 1.00 (0.99–1.01)	X^2^(1) = 0.19
**Household income**, %			X^2^(2) = 0.62
<CAD 50,000	23.4% 1.0 Reference	22.0% 0.78 (0.32–1.93)	
CAD 50,000–CAD 100,000	46.3% 1.0 Reference	41.5% 0.74 (0.35–1.59)	
>CAD 100,000	30.4% 1.0 Reference	36.6% 1.0 Reference	
**Currently in a relationship**, %			X^2^(1) = 0.25
No	1.3% 1.0 Reference	2.2% 1.79 (0.18–17.58)	
Yes	98.7% 1.0 Reference	97.8% 1.0 Reference	

* *p* < 0.05, ** *p* < 0.01; *** *p* < 0.001 ^1^ Lower score indicates worse quality of life.

**Table 3:** Multivariate logistic regression modelling the relationship between mental disorder and social/family, emotional, functional and spiritual well-being, urinary function, treatment modality and demographic variables in a sample of men with a history of prostate cancer diagnosis from the baseline cycle of a Quality-of-Life Maritimes Survey administered between 2017 and 2021, *n* = 284.

Predictors	Screening Negative for Mental Disorder (*n* = 239) 1.0 Reference	Screening Positive for Mental Disorder (*n* = 45) aOR (95% CI) ^a^ *aOR_MI_ (95% CI) ^b^*	Wald X^2 a^ *Wald X^2 b^*
			X^2^(12) = 90.23 ***
**Urinary tract symptoms**, **I-PSS**			
**Moderate or severe vs. None to mild (Reference)**	1.0 Reference	3.02 (1.10–8.32) * 3.27 (1.38–7.75) *	X^2^(1) = 4.56 * X^2^(1) = 7.27 *
**Social/family well-being** ^1^, **FACT-P**	1.0 Reference	0.94 (0.83–1.05) *0.93 (0.84–1.03)*	X^2^(1) = 0.94 *X^2^(1) = 2.17*
**Emotional well-being ^1^**, **FACT-P**	1.0 Reference	0.81 (0.69–0.96) * 0.87 (0.76–0.98) *	X^2^(1) = 5.86 * X^2^(1) = 4.52 *
**Functional well-being ^1^**, **FACT-P**	1.0 Reference	1.00 (0.87–1.15) *1.02 (0.90–1.14)*	X^2^(1) = 0.01 *X^2^(1) = 0.06*
**Spiritual well-being ^1^**, **FACIT-Sp**	1.0 Reference	0.88 (0.81–0.96) ** 0.92 (0.86–0.99) *	X^2^(1) = 8.80 ** X^2^(1) = 5.66 *
**Treatment Modality**			X^2^(2) = 5.91 * X^2^(2) = 7.64 *
Active surveillance vs. Radiation or Hormone therapy alone, or both (Reference)	1.0 Reference	5.87 (1.32–26.13) * 4.95 (1.55–15.82) *	
Surgery (alone or plus other forms of treatment) vs. Radiation or Hormone therapy alone, or both (Reference)	1.0 Reference	4.21 (1.07–16.51) * 3.95 (1.15–13.63) *	
**Age**	1.0 Reference	1.01 (0.94–1.08) *1.02 (0.96–1.08)*	X^2^(1) = 0.06 *X^2^(1) = 0.45*
**Survivorship time (months) from diagnosis**	1.0 Reference	1.00 (0.99–1.01) *1.00 (0.99–1.01)*	X^2^(1) = 0.69 *X^2^(1) = 0.40*
**Household income**			X^2^(2) = 4.78 *X^2^(2) = 4.23*
<CAD 50,000 vs. >CAD 100,000 (Reference)	1.0 Reference	0.27 (0.07–1.07) *0.35 (0.11–1.09)*	
CAD 50,000–CAD 100,000 vs. >CAD 100,000 (Reference)	1.0 Reference	0.32 (0.09–1.04) *0.43 (0.17–1.13)*	
**Currently in a relationship**			X^2^(1) = 0.70 *X^2^(1) = 2.84*
No vs. Yes (Reference)	1.0 Reference	0.24 (0.01–6.78) *0.30 (0.07–1.22)*	

* *p* < 0.05, ** *p* < 0.01; *** *p* < 0.001; aOR—adjusted odds ratios; ^a^ original data; ^b^ multiple imputations pooled data based on 41 imputations. ^1^ Lower scores indicates worse quality of life.

## Data Availability

Data from this study are available to researchers through a data access process.
